# Translating the pathophysiology of sepsis-associated acute kidney injury: From bench to bedside

**DOI:** 10.1016/j.jointm.2026.08.005

**Published:** 2026-09-01

**Authors:** Mengfei Lu, Qi Zhang, Mingmin Pang, Haoyu Wang, Miaosa Zhang, Yihui Li, Hao Wang

**Affiliations:** 1 Department of Critical Care Medicine, Qilu Hospital, Innovation Research Center for Sepsis and Multiple Organ Injury, Shandong University, Jinan, Shandong, China; 2 Department of Critical Care Medicine, Shandong University Qilu Hospital Dezhou Hospital, Dezhou, Shandong, China

**Keywords:** Sepsis-associated acute kidney injury, Inflammatory dysregulation, Microcirculatory dysfunction, Regulated cell death, Organ crosstalk, Biomarkers

## Abstract

Sepsis-associated acute kidney injury (SA-AKI) is a major complication in the intensive care unit (ICU), associated with increased mortality and progression to chronic kidney disease (CKD) and end-stage renal disease (ESRD). This review summarizes current advances in the definition, epidemiology, pathophysiology, diagnosis, experimental modeling, and therapeutic strategies of SA-AKI, with emphasis on translational challenges. The conceptual framework of SA-AKI has evolved from global renal hypoperfusion to a multifactorial, predominantly non-ischemic process characterized by inflammatory dysregulation, microcirculatory dysfunction, and adaptive metabolic reprogramming. Tubular metabolic shutdown is increasingly recognized as a protective yet maladaptive response contributing to functional decline. Regulated cell death pathways, including pyroptosis and ferroptosis, further drive tubular injury. In parallel, organ crosstalk – particularly along the lung–gut–heart–kidney axis – amplifies renal dysfunction during sepsis. Recent diagnostic advances are highlighted, including the 2023 Acute Disease Quality Initiative (ADQI) consensus and emerging biomarkers reflecting distinct pathophysiological domains: tubular injury (neutrophil gelatinase-associated lipocalin [NGAL] and kidney injury molecule-1 [KIM-1]), cell cycle arrest (Tissue Inhibitor of Metalloproteinases-2[TIMP-2]·insulin-like growth factor-binding protein 7 [IGFBP7]), and endothelial dysfunction (angiopoietin-2 [Angpt-2] and the tyrosine kinase with immunoglobulin-like and EGF-like domains 2 receptor [Tie2]), supporting early risk stratification beyond serum creatinine (sCr). Experimental models ranging from endotoxemia and cecal ligation and puncture to two-hit and comorbidity-based paradigms are critically appraised. Finally, current and investigational therapies – including hemodynamic optimization, extracorporeal cytokine removal, mitochondria-targeted interventions, and regenerative approaches – are discussed in the context of persistent barriers to clinical translation.

## Introduction

Sepsis stands as the primary driver of multiple organ dysfunction and mortality within the intensive care unit (ICU).^[^^1^^]^ Among its complications, sepsis-associated acute kidney injury (SA-AKI) has emerged as a critical clinical challenge requiring urgent intervention in the field of critical care medicine.^[^[1], [2], [3], [4], [5]^]^ Current epidemiological data indicate that sepsis affects approximately 19 million patients globally each year, with two-thirds developing SA-AKI, resulting in an annual incidence exceeding 11 million cases.^[^^1^^,^^2^^,^^5^^,^^6^^]^ This complication not only exacerbates the severity of the illness but also leads to profoundly poor outcomes. The in-hospital mortality rate for septic patients with SA-AKI is 1.48 times higher than those without acute kidney injury (AKI). Furthermore, the average length of hospital stay reaches 37 days (compared with 21 days for non-septic AKI), and nearly 25% of these patients require renal replacement therapy (RRT).^[^^7^^]^ Even for those who survive the acute phase, the risk of progressing to chronic kidney disease (CKD) or end-stage renal disease (ESRD) increases threefold. The 3-year mortality rate stands at 44%, significantly higher than the 28% observed in patients with recovered renal function.^[^^8^^,^^9^^]^ This trajectory underscores a vicious cycle of “acute injury, chronic progression, and long-term adverse outcomes.” The clinical recognition and management of SA-AKI currently face multiple challenges. In terms of diagnosis, the current Kidney Disease: Improving Global Outcomes (KDIGO) criteria rely on increased serum creatinine (sCr) levels and decreased urine output.^[^^7^^,^^10^^]^ However, in sepsis, muscle hypoperfusion leads to reduced creatinine production, and extensive fluid resuscitation induces a dilution effect, resulting in approximately 30% of early SA-AKI cases being missed.^[^^11^^,^^12^^]^ Additionally, sepsis and AKI have a “bidirectional effect” – 40% of AKI patients subsequently develop sepsis,^[^^13^^,^^14^^]^ and sepsis can directly or indirectly damage the kidneys, making it difficult to establish a clear causal relationship. At the pathological mechanism level, the traditional view of “global renal hypoperfusion leading to acute tubular necrosis (ATN)” has been overturned.^[^^15^^]^ Studies have shown that 25% of SA-AKI patients have normal or increased renal blood flow,^[^[16], [17], [18]^]^ and autopsy findings show that <5% of renal tubular epithelial cells (TECs) exhibit necrosis.^[^^19^^]^ The current unified theory of sepsis-induced AKI (SI-AKI) emphasizes non-ischemic mechanisms, such as uncontrolled inflammation, microcirculatory disturbances, and cellular metabolic reprogramming.^[^^20^^]^ Regarding treatment, there is still no specific drug for SA-AKI, and interventions focus on infection control, fluid resuscitation, and avoiding nephrotoxic agents. However, excessive fluid overload increases the risk of renal interstitial edema, and the timing of RRT initiation (early *vs*. delayed) remains controversial and has yet to reach a consensus,^[^^21^^]^ limiting consistency in clinical decision-making.

A recent review by Hato and Dagher^[^^22^^]^ provided a focused account of the molecular timeline and dynamic cellular responses underlying SA-AKI. By contrast, the present review adopts a broader bench-to-bedside perspective by integrating disease definition, epidemiology, mechanistic networks, biomarkers, experimental models, and therapeutic translation.

## Definition and Epidemiology of SA-AKI

SA-AKI is defined by the 2023 Acute Disease Quality Initiative (ADQI) consensus as meeting two criteria with a clear temporal relationship: sepsis diagnosis based on Sepsis-3 criteria (organ dysfunction with Sequential Organ Failure Assessment [SOFA] score ≥2) and AKI diagnosis following KDIGO criteria (increase in sCr ≥0.3 mg/dL within 48 h, or a 50% increase within 7 days, or urine output <0.5 mL/(kg·h) for 6–12 h). AKI must occur within 7 days of sepsis diagnosis.^[^^23^^,^^24^^]^ SA-AKI is categorized into early (within 48 h of sepsis diagnosis, 63.9%–97%) and late (48 h to 7 days, 36.1%–40%) onset, with a subphenotype of SI-AKI that excludes indirect factors such as nephrotoxic drugs and intra-abdominal hypertension. Biomarkers can aid in clinical diagnosis.^[^^24^^]^ The 2023 ADQI consensus not only updated the diagnostic framework of SA-AKI but also emphasized that SA-AKI is a heterogeneous clinical syndrome rather than a single disease entity. The consensus highlighted the importance of integrating clinical context, temporal evolution, and biological heterogeneity into future mechanistic studies and therapeutic development, thereby promoting precision medicine approaches and improving the translational relevance of preclinical research.^[^^24^^]^

SA-AKI is one of the most common complications in ICU sepsis patients, with its incidence influenced by diagnostic criteria and regional differences.^[^^25^^]^ The incidence in ICU sepsis patients is 46.6%, while for all adult ICU admissions it is 15.8%.^[^^24^^]^ Global estimates of annual SA-AKI incidence range from 6 million to 10 million cases.^[^^26^^]^ Due to varying definitions, reported incidences have varied across studies, but with the ADQI 2023 consensus, rates across regions are more comparable. For example, a multicenter study in 24 European countries found a 54% incidence of AKI in sepsis patients,^[^^27^^]^ while a retrospective study in China reported a 47.1% incidence.^[^^28^^]^ In pediatric SA-AKI, incidence in ICU sepsis patients is 34%–50%, with infants and young children at higher risk for severe progression due to immature kidney development.^[^^29^^]^

Prognostically, SA-AKI worsens outcomes significantly. In-hospital mortality is 25%–41%, 1.48 times higher than sepsis alone (11.2%) and 1.59 times higher than AKI alone. The incidence of major kidney adverse events at discharge is 37.7%. Late-onset SA-AKI has higher mortality than early-onset, with mortality doubling in patients with septic shock. Long-term, 21%–79% of surviving SA-AKI patients progress to CKD within 1 year, with a 3-year mortality rate of 44% for unresolved kidney function, compared with 28% for those with restored function.^[^^30^^]^

## Pathophysiological Mechanisms of SA-AKI

The pathogenesis of SA-AKI mainly includes systemic inflammatory responses induced by pathogen-associated molecular patterns (PAMPs)/damage-associated molecular patterns (DAMPs), microcirculatory dysfunction accompanied by endothelial injury and perfusion–oxygenation mismatch, metabolic shutdown and mitochondrial dysfunction in renal TECs, as well as regulated cell death such as pyroptosis and ferroptosis, all of which aggravate renal injury (Figure 1).

**Figure 1:**
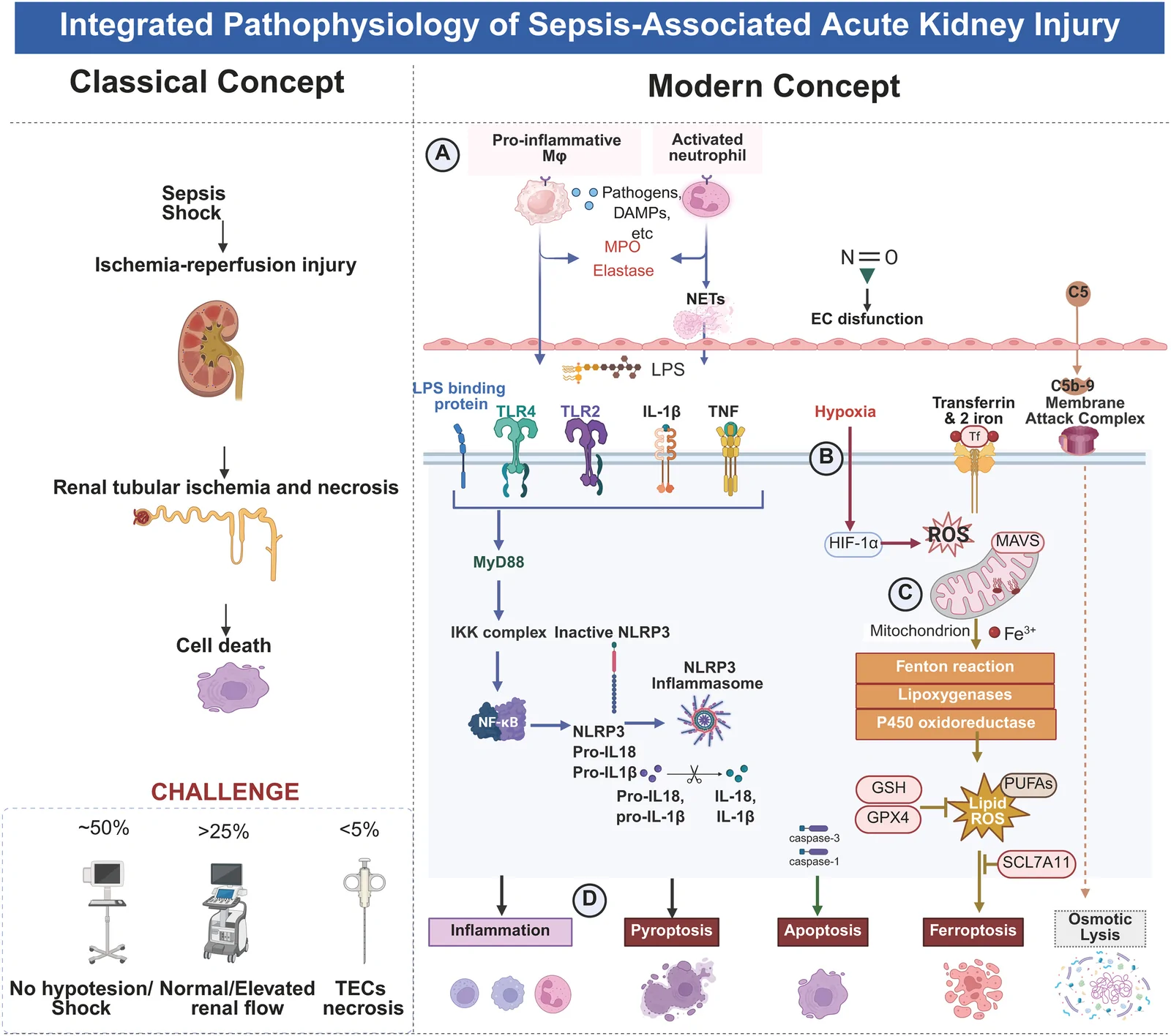
Integrated pathophysiology of SA-AKI. A: Systemic inflammation: PAMPs (e.g., LPS) and DAMPs activate pattern recognition receptors (e.g., TLR4) on renal TECs and resident immune cells, triggering the NF-κB and NLRP3 inflammasome pathways. B: Microcirculatory dysfunction: Endothelial injury leads to the shedding of the glycocalyx, microthrombi formation, and leukocyte adhesion, resulting in localized perfusion-oxygenation mismatch. C: Metabolic reprogramming: TECs undergo a “metabolic shutdown,” shifting from OXPHOS to inefficient glycolysis, characterized by mitochondrial swelling and reduced ATP production. D: Regulated cell death: Novel pathways including GSDMD-mediated pyroptosis and iron-dependent ferroptosis drive the irreversible loss of TECs and amplify local inflammation. DAMPs, Damage-associated molecular patterns; EC, Endothelial Cell; GPX4, Glutathione peroxidase 4; GSDMD, Gasdermin D; GSH, Glutathione; HIF-1α, Hypoxia-Inducible Factor 1-Alpha; IKK, IκB Kinase; IL-18, Interleukin-18; IL-1β, Interleukin-1β; LPS, Lipopolysaccharide; MAVS, Mitochondrial Antiviral Signaling protein; MPO, Myeloperoxidase; NETs, Neutrophil extracellular traps; NF-κB, ar factor kappa B; NLRP3, NOD-like receptor protein 3; PAMPs, Pathogen-associated molecular patterns; PUFAs, Polyunsaturated fatty acids; OXPHOS, Oxidative phosphorylation; ROS, Reactive oxygen species; SA-AKI, Sepsis-associated acute kidney injury; SLC7A11, Solute Carrier Family 7 Member 11; TECs, Tubular epithelial cells; TLR2, Toll-like receptor 2; TLR4, Toll-like receptor 4; TNF, Tumor necrosis factor.

### Traditional approach: centered on hemodynamics

Early understanding of the pathological mechanism of SA-AKI focused on “renal ischemia-reperfusion injury.” The core view held that systemic hemodynamic disorders caused by sepsis were the primary cause of renal injury, and “renal tubular ischemic necrosis induced by macrohypoperfusion” was regarded as the main mechanism. However, subsequent studies have challenged this notion: renal blood flow is normal or even increased in approximately 25% of SA-AKI patients,^[^^31^^,^^32^^]^ fewer than 5% of renal TECs show necrosis in autopsies.^[^^19^^]^ SA-AKI can still occur in some patients without obvious hypotension or shock,^[^^33^^]^ and some animal experiments have failed to induce AKI even in the setting of hemorrhagic shock.^[^^34^^,^^35^^]^ These findings suggest that SA-AKI is not driven by a single pathogenic mechanism but rather arises from the dynamic interplay of multiple pathological processes, including dysregulated inflammation, endothelial injury, microcirculatory dysfunction, metabolic reprogramming, and regulated cell death. These interconnected mechanisms reinforce one another, forming a self-amplifying positive feedback network that collectively determines the severity of renal injury and its subsequent repair outcomes. Therefore, elucidating the crosstalk among these pathogenic pathways is essential for the development of novel therapeutic strategies.

### Inflammatory response

Inflammatory dysregulation is a major driver of SA-AKI and serves as a central link among endothelial injury, microcirculatory dysfunction, metabolic reprogramming, and regulated cell death.^[^^24^^]^ However, as implied by the new definition of sepsis, a dysregulated host response contributes substantially to organ dysfunction and adverse outcomes in sepsis.^[^^3^^,^^36^^]^ Sepsis is essentially an “infection-induced host immune disorder.” As a highly perfused and metabolically active organ, the kidney is one of the main target organs of immune-inflammatory injury.^[^^37^^]^ During sepsis, signaling molecules such as bacterial lipopolysaccharide (LPS) and high-mobility group box 1 (HMGB1) released by necrotic cells activate downstream signaling pathways through pattern recognition receptors (e.g., toll-like receptor 4 [TLR4]) on the surface of intrinsic renal cells.^[^^38^^,^^39^^]^ For instance, renal TECs highly express TLR2/4, and their activation promotes the release of proinflammatory cytokines such as tumor necrosis factor-α (TNF-α) and interleukin-6 (IL-6) via the nuclear factor-kappa B (NF-κB) pathway,^[^[40], [41], [42]^]^ directly damaging renal TECs. Activation of the NOD-like receptor protein 3 (NLRP3) inflammasome mediates the mature release of interleukin-1β (IL-1β) and interleukin-18 (IL-18) through caspase-1, exacerbates local inflammatory infiltration, and simultaneously induces pyroptosis of renal tubular cells.^[^^43^^]^

In addition to resident renal cells, circulating immune cells constitute another major source of sustained inflammatory amplification. Following the onset of sepsis, neutrophils and monocytes/macrophages are recruited to the kidney via the circulation, where they contribute to host defense through pathogen phagocytosis.^[^[44], [45], [46]^]^ However, excessive activation of these immune cells also leads to the release of reactive oxygen species (ROS), myeloperoxidase (MPO), and proteolytic enzymes, such as neutrophil elastase, which directly damage the tubular basement membrane and renal TECs.^[^^47^^]^ Moreover, dysregulated macrophage polarization, characterized by a predominance of pro-inflammatory M1 macrophages, further amplifies the inflammatory response while impairing tubular repair and regeneration.^[^^48^^]^ Recent studies have further demonstrated that neutrophil extracellular traps (NETs) not only facilitate pathogen trapping but also induce endothelial injury, activate the coagulation cascade, and promote immunothrombosis.^[^^49^^]^ These findings identify NETs as a critical mechanistic link between excessive inflammation and microcirculatory dysfunction, providing new insights into the persistent progression of SA-AKI. Complement overactivation further exacerbates renal injury during sepsis. The complement fragments C3a and C5a recruit inflammatory cells and increase vascular permeability, thereby intensifying renal inflammation and tissue damage.^[^^50^^,^^51^^]^ In addition, insertion of the membrane attack complex (C5b-9) into the plasma membrane of TECs induces cell lysis, representing one of the characteristic pathological features observed in the kidneys of patients with SA-AKI.^[^^52^^,^^53^^]^

Importantly, inflammatory dysregulation acts as a central hub coordinating multiple pathogenic processes. Persistent inflammation promotes endothelial activation, glycocalyx degradation, and coagulation abnormalities, thereby impairing renal microvascular perfusion. Meanwhile, inflammation-induced ROS overproduction and mitochondrial dysfunction drive metabolic reprogramming and multiple forms of regulated cell death. The subsequent release of DAMPs further reactivates inflammatory signaling, establishing a self-perpetuating positive feedback loop that sustains kidney injury. Collectively, these findings indicate that inflammatory dysregulation is not only the initiating event in SA-AKI but also a pivotal nexus linking microcirculatory dysfunction, metabolic abnormalities, and cell fate determination.^[^^54^^]^

### Endothelial cell injury and microcirculatory dysfunction

Persistent inflammation not only directly injures renal TECs but also disrupts endothelial homeostasis, thereby driving renal microcirculatory dysfunction.^[^^55^^]^ Continuous stimulation of endothelial cells by pro-inflammatory cytokines, such as TNF-α and IL-1β, together with PAMPs and DAMPs, promotes glycocalyx degradation and upregulates endothelial adhesion molecules. These changes facilitate leukocyte adhesion, platelet aggregation, and microthrombus formation, leading to obstruction of the glomerular and outer medullary microvasculature.^[^^56^^,^^57^^]^ Loss of glycocalyx integrity further promotes inflammatory cell infiltration into the renal interstitium while exposing endothelial adhesion molecules that enhance neutrophil and platelet adhesion, thereby amplifying local inflammation and immunothrombosis. Collectively, these events culminate in profound renal microcirculatory heterogeneity.^[^^58^^]^ Emerging evidence suggests that microcirculatory dysfunction is not synonymous with global renal hypoperfusion. Even after restoration of total renal blood flow, persistent capillary “no-reflow” may occur, particularly within the highly oxygen-demanding outer medulla, resulting in sustained regional hypoperfusion and tissue hypoxia. This concept has fundamentally shifted the current understanding of SA-AKI pathophysiology from one centered on global renal ischemia to one emphasizing microvascular dysfunction. In parallel, excessive nitric oxide (NO) reacts with superoxide to generate peroxynitrite, impairing endothelium-dependent vasodilation and disrupting renal autoregulation.^[^^59^^]^ The resulting reduction in tissue perfusion exacerbates tubular hypoxia, while mitochondrial dysfunction further aggravates cellular energy depletion.^[^^60^^]^ Meanwhile, aberrant activation of the coagulation cascade, suppression of endogenous anticoagulant pathways (including the protein C system), and impaired fibrinolysis collectively promote diffuse intrarenal microvascular thrombosis, thereby worsening renal parenchymal injury.^[^^61^^]^ Notably, sustained microvascular hypoperfusion, oxidative stress, and the inflammatory milieu collectively suppress mitochondrial oxidative phosphorylation (OXPHOS), forcing TECs to shift from their highly efficient fatty acid oxidation (FAO)-dependent metabolic program toward a low-energy metabolic state that supports short-term cell survival. However, this adaptive response comes at the expense of ion transport, tubular reabsorption, and epithelial repair. When inflammation and microvascular hypoperfusion persist, the initial metabolic adaptation progressively evolves into a state of persistent metabolic shutdown, ultimately promoting multiple forms of regulated cell death and renal dysfunction. Therefore, microcirculatory dysfunction represents a critical pathological node linking inflammatory dysregulation to metabolic reprogramming and provides an important mechanistic basis for understanding the transition of SA-AKI from reversible functional impairment to irreversible structural kidney injury.^[^^54^^]^

### Metabolic reprogramming and mitochondrial dysfunction: “metabolic shutdown” in renal tubular cells

The most profound consequence of microcirculatory dysfunction is its ability to drive metabolic reprogramming in renal TECs. Emerging evidence suggests that the metabolic abnormalities observed in SA-AKI do not simply reflect energy depletion but rather represent an adaptive response aimed at preserving cell survival, a phenomenon often referred to as metabolic shutdown.^[^^54^^,^^62^^]^ Although initially protective, this metabolic adaptation compromises cellular function and exacerbates injury by promoting lactate accumulation and oxidative stress.^[^^63^^]^ Under persistent hypoxia and inflammatory stimulation, TECs undergo phenotypic alterations, with a subset acquiring myofibroblast-like characteristics that contribute to renal interstitial fibrosis.^[^^64^^]^

Mitochondrial dysfunction represents the central event underlying metabolic shutdown. Renal TECs are highly enriched in mitochondria and rely predominantly on FAO and OXPHOS to meet their substantial energy demands. Sustained inflammation, oxidative stress, and regional hypoxia collectively disrupt mitochondrial integrity, leading to cristae fragmentation, mitochondrial swelling and vacuolization, impaired Adenosine triphosphate(ATP) production, and progressive cellular energy failure, thereby accelerating both necrotic and apoptotic cell death.^[^^65^^]^ The nicotinamide adenine dinucleotide (NAD^+^)-dependent sirtuin signaling axis constitutes a key molecular regulator of metabolic homeostasis and mitochondrial quality control. Persistent inflammation and oxidative stress reduce intracellular NAD^+^ availability, thereby attenuating Sirtuin‑3(SIRT3)-mediated deacetylation of manganese superoxide dismutase (MnSOD) and weakening mitochondrial antioxidant defenses. Concurrently, inhibition of the Sirtuin‑1(SIRT1)/Peroxisome proliferator‑activated receptor‑gamma coactivator‑1‑alpha(PGC-1α) signaling pathway suppresses mitochondrial biogenesis, impairs FAO, and further compromises OXPHOS capacity. Damaged mitochondria subsequently release excessive ROS and mitochondrial DNA (mtDNA), which act as danger-associated molecular patterns to amplify inflammatory signaling and aggravate metabolic dysfunction, establishing a self-perpetuating positive feedback loop between inflammation and metabolic derangement. Increasing evidence suggests that restoration of NAD^+^ homeostasis, activation of SIRT1 and SIRT3, and improvement of mitochondrial quality control may reverse metabolic shutdown and facilitate renal recovery, making the NAD^+^/Sirtuin signaling axis an emerging therapeutic target for metabolic intervention in SA-AKI.^[^^66^^]^

Beyond mitochondrial dysfunction, endoplasmic reticulum stress (ERS) and disrupted inter-organelle communication further aggravate metabolic imbalance. Persistent inflammation activates the unfolded protein response (UPR), which initially serves as an adaptive mechanism to restore proteostasis. However, when ER stress exceeds the cellular adaptive capacity, prolonged UPR signaling triggers apoptosis through C/EBP homologous protein (CHOP)-dependent pathways.^[^^67^^,^^68^^]^ Meanwhile, dysfunction of mitochondria-associated membranes (MAMs) disrupts intracellular calcium homeostasis, promoting sustained opening of the mitochondrial permeability transition pore (mPTP), thereby exacerbating mitochondrial injury, impairing energy metabolism, and amplifying organelle dysfunction.^[^[69], [70], [71]^]^ Collectively, these alterations compromise cellular repair mechanisms, hinder the efficient clearance of damaged mitochondria, and ultimately trap TECs in a persistent state of low-energy metabolism and heightened oxidative stress.^[^^72^^]^

Together, these interconnected metabolic disturbances drive TECs toward irreversible injury, thereby promoting the transition from AKI to CKD following sepsis.

### Regulated cell death: a common terminal pathway linking inflammation and metabolic dysregulation

Apoptosis was traditionally considered to be the predominant mode of cell death in SA-AKI.^[^^73^^]^ However, accumulating experimental and clinical evidence has established pyroptosis as a central driver of renal injury in SA-AKI, while necroptosis and ferroptosis further exacerbate tubular damage through distinct yet interconnected mechanisms.^[^[74], [75], [76]^]^ More recently, the concept of PANoptosis has emerged, proposing that multiple regulated cell death pathways are coordinately activated during sepsis and collectively contribute to kidney injury.^[^^77^^]^

Pyroptosis is an inflammatory form of regulated cell death mediated by caspase-1/4/5/11 signaling. During sepsis, LPS activates the canonical pathway through TLR4-dependent NLRP3 inflammasome assembly or directly engages caspase-4/5 in humans and caspase-11 in mice via the non-canonical pathway. Activated inflammatory caspases cleave gasdermin D (GSDMD), generating membrane pores that induce swelling and lytic death of renal TECs, accompanied by the release of IL-1β and IL-18. Increased expression of GSDMD and caspase-1 has been consistently observed in renal tissues from patients with SA-AKI and correlates with disease severity and mortality, whereas genetic deletion of GSDMD markedly attenuates tubular injury in experimental models.^[^[78], [79], [80]^]^

Necroptosis is a programmed form of necrotic cell death governed by the receptor-interacting protein kinase (RIPK) signaling axis. Following TNF-α engagement of TNF receptor 1 (TNFR1), inhibition or insufficient activation of caspase-8 permits Receptor-interacting serine/threonine-protein kinase 1(RIPK1) and Receptor-interacting serine/threonine-protein kinase 3(RIPK3) to interact through their RIP homotypic interaction motif (RHIM), leading to formation of the necrosome. RIPK3 subsequently phosphorylates mixed lineage kinase domain-like protein (MLKL), whose oligomerization and translocation to the plasma membrane result in membrane disruption, release of intracellular contents and DAMPs, and amplification of sterile inflammation. In SA-AKI, persistent inflammatory signaling mediated by TLR4, TNF-α, and related pathways sustains activation of the RIPK1/RIPK3/MLKL cascade, thereby promoting necroptotic death of TECs and aggravating renal inflammation and functional impairment.^[^[81], [82], [83]^]^

Ferroptosis is an iron-dependent form of regulated cell death characterized by excessive lipid peroxidation. During sepsis, IL-6 stimulates hepatic hepcidin production, while endothelial injury promotes intrarenal accumulation of labile ferrous iron (Fe^2+^). Through the Fenton reaction, excess iron generates ROS that preferentially oxidize polyunsaturated fatty acids (PUFAs) within membrane phospholipids. Simultaneously, sepsis suppresses glutathione peroxidase 4 (GPX4), the principal enzyme responsible for detoxifying lipid hydroperoxides, thereby accelerating the accumulation of toxic lipid peroxidation products, including 4-hydroxynonenal (4-HNE), ultimately leading to ferroptotic cell death. Pharmacological inhibition of ferroptosis using iron chelators, such as deferoxamine, has been shown to alleviate renal injury in experimental SA-AKI.^[^^84^^,^^85^^]^

PANoptosis is a recently recognized inflammatory form of regulated cell death that integrates pyroptosis, apoptosis, and necroptosis into a coordinated cell death program rather than representing any single pathway alone. Distinct pathogens and danger signals trigger the assembly of multiprotein PANoptosomes through innate immune sensors, including Z-DNA binding protein 1(ZBP1), Absent in melanoma 2(AIM2), RIPK1, and NACHT, LRR and PYD domains-containing protein 12(NLRP12). These complexes recruit key signaling molecules such as apoptosis-associated speck-like protein containing a CARD (ASC), Fas-associated death domain protein (FADD), caspase-8, RIPK3, and caspase-1, thereby simultaneously activating GSDMD-dependent pyroptosis, caspase-3/7-mediated apoptosis, and MLKL-dependent necroptosis.^[^^77^^,^^86^^,^^87^^]^ Increasing evidence indicates that extensive crosstalk exists among multiple regulated cell death pathways during sepsis, collectively amplifying inflammation and organ injury. Consequently, PANoptosis has emerged as a promising conceptual framework for understanding the complex pathogenesis of SA-AKI and may provide novel therapeutic opportunities for targeting multiple cell death pathways simultaneously.

### Organ-interaction-mediated renal injury in sepsis

The pathogenesis of sepsis-associated kidney injury is addressed via the lung–kidney, gut–kidney, and heart–kidney axes, as illustrated in Figure 2.

**Figure 2:**
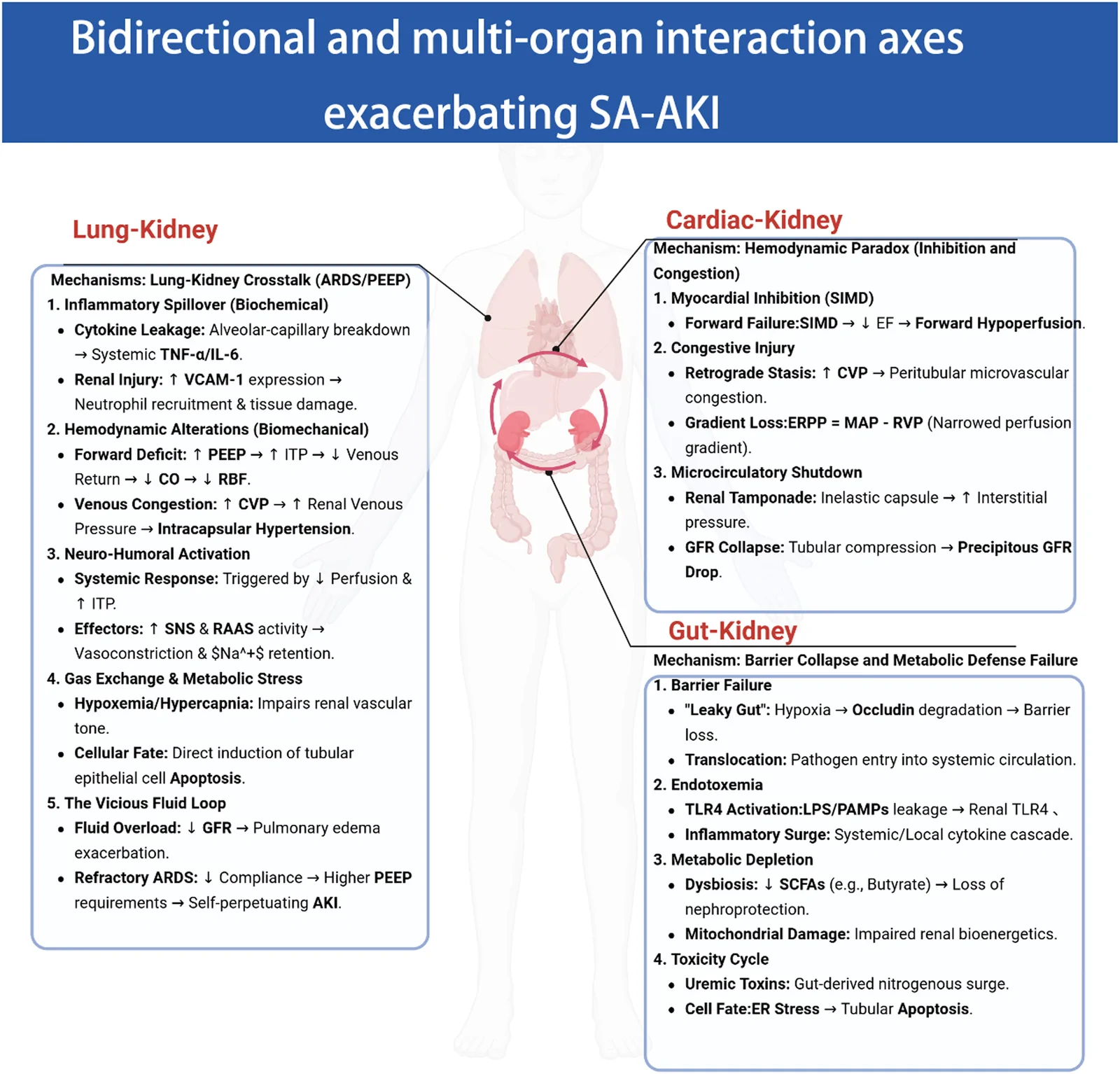
Bidirectional and multi-organ interaction axes exacerbating SA-AKI. Sepsis-induced kidney injury is part of a systemic network involving multiple organ axes. A: Lung–kidney axis: Systemic cytokine release from injured lungs (e.g., ARDS) affects the kidneys, while high levels of PEEP during mechanical ventilation increase intrathoracic pressure, impair venous return, and reduce renal perfusion. B: Heart–kidney axis (type 5 cardiorenal syndrome): SIMD reduces CO, while elevated CVP causes renal venous congestion and interstitial edema, significantly decreasing the glomerular filtration gradient. C: Gut–kidney axis: Breakdown of the intestinal barrier (“leaky gut”) allows translocation of bacteria and endotoxins into the circulation. Simultaneously, depletion of microbiota-derived SCFAs weakens the kidney’s antioxidant defenses. AKI, Acute kidney injury; ARDS, Acute respiratory distress syndrome; CO, Cardiac output; CVP, Central venous pressure; EF, Ejection Fraction; ERS, Endoplasmic reticulum stress; GFR, Glomerular filtration rate; IL-6, Interleukin-6; LPS, Lipopolysaccharide; ITP, Intrathoracic Pressure; MAP, Mean arterial pressure; PAMPs, Pathogen-associated molecular patterns; PEEP, Positive end-expiratory pressure; RAAS, Renin-Angiotensin-Aldosterone System; RBF, Renal Blood Flow; RVP, Renal venous pressure; SA-AKI, Sepsis-associated acute kidney injury; SCFAs, Short-chain fatty acids; SIMD, Sepsis-induced myocardial dysfunction; SNS, Sympathetic Nervous System; TLR4, Toll-like receptor 4; TNF-α, Tumor necrosis factor-α; VCAM-1, Vascular cell adhesion molecule-1.

#### Lung–kidney axis: from cytokine spillover to biomechanical injury

Within the network of sepsis-induced multiple organ dysfunction, the lung and kidney are tightly interconnected through two complementary mechanisms: inflammatory mediator spillover and biomechanical stress transmission.^[^^88^^]^ When severe pulmonary infection progresses to acute respiratory distress syndrome (ARDS), disruption of the alveolar–capillary barrier transforms the injured lung into a major source of systemic inflammatory mediators. Among these, the HMGB1-TLR4/receptor for advanced glycation end products (RAGE) signaling axis has emerged as a pivotal molecular link between pulmonary inflammation and secondary kidney injury. HMGB1 released from the injured lung activates TLR4/RAGE signaling in the kidney, thereby promoting NF-κB activation and the production of pro-inflammatory cytokines. Concurrently, large amounts of TNF-α, IL-1β, and IL-6 enter the systemic circulation and reach the renal microvasculature, where they activate glomerular endothelial cells, upregulate vascular adhesion molecules such as vascular cell adhesion molecule-1 (VCAM-1), and promote neutrophil recruitment into the renal interstitium. Activated neutrophils subsequently release ROS, initiating a secondary inflammatory cascade within the kidney.^[^^89^^,^^90^^]^ In parallel, NETs further aggravate endothelial injury and promote immunothrombosis, thereby exacerbating renal microcirculatory dysfunction. Consequently, HMGB1, NETs, and the complement/C5a signaling pathway have emerged as promising therapeutic targets within the lung–kidney axis, and their pharmacological inhibition may attenuate remote organ inflammation and tissue injury.^[^^91^^]^

Beyond inflammatory signaling, mechanical ventilation itself represents an important source of organ crosstalk through biomechanical stress. Positive end-expiratory pressure (PEEP), while essential for alveolar recruitment and oxygenation in patients with ARDS, exerts profound hemodynamic effects on renal perfusion. Elevated intrathoracic pressure compresses the vena cava and reduces venous return, resulting in decreased cardiac output (CO) and diminished forward renal perfusion pressure. More importantly, impaired venous drainage increases renal venous pressure (RVP), leading to renal venous congestion and elevated intracapsular pressure, both of which further compromise renal microcirculation.^[^^92^^]^ Thus, the convergence of inflammatory mediator spillover and ventilation-induced biomechanical stress constitutes a dual-hit mechanism that accelerates renal dysfunction following sepsis-associated lung injury.

#### Gut–kidney axis: barrier disruption, microbial translocation, and metabolic failure

During sepsis, the gut–kidney axis illustrates the progression from localized mucosal injury to systemic metabolic dysregulation.^[^^93^^]^ Sepsis-induced hemodynamic redistribution preferentially compromises intestinal perfusion, resulting in ischemic injury and necrosis of the intestinal epithelium. This process is accompanied by downregulation of tight junction proteins, including occludin, leading to disruption of the intestinal barrier. The resulting “leaky gut” permits large-scale translocation of LPS and other PAMPs into the circulation, where they reach the kidney through the portal and systemic circulation and activate TLR4-dependent inflammatory signaling in TECs.^[^^94^^,^^95^^]^

Recent studies have further demonstrated that gut microbiota-derived metabolites not only regulate host immunity but also directly influence renal redox homeostasis. Among these metabolites, short-chain fatty acids (SCFAs), particularly butyrate, propionate, and acetate, exert renoprotective effects by activating the G protein-coupled receptors G protein-coupled receptor 41(GPR41) and G protein-coupled receptor 43(GPR43) while simultaneously inhibiting histone deacetylases (HDACs).^[^^96^^]^ These actions enhance nuclear factor erythroid 2-related factor 2 (Nrf2)-dependent antioxidant signaling, leading to upregulation of antioxidant enzymes such as heme oxygenase-1 (HO-1) and GPX4, thereby attenuating lipid peroxidation, suppressing ferroptosis, and preserving redox homeostasis in TECs. In addition, SCFAs improve intestinal barrier integrity, reduce systemic translocation of gut-derived endotoxins, including LPS, and attenuate activation of the TLR4/NF-κB signaling pathway, thereby limiting systemic inflammation. Consequently, restoration of gut microbial homeostasis, supplementation with SCFAs, and therapeutic targeting of the GPR41/GPR43–Nrf2 signaling axis have emerged as promising strategies for modulating the gut–kidney axis in SA-AKI.^[^^97^^]^

#### Heart–kidney axis: hemodynamic paradox driven by myocardial depression and venous congestion

Sepsis-induced myocardial injury represents another important driver of renal dysfunction through the heart–kidney axis. Injured cardiomyocytes release large amounts of DAMPs, which may contribute to activate the TLR4/NF-κB signaling pathway in the kidney and promote sustained production of pro-inflammatory cytokines, including TNF-α and IL-1β, thereby exacerbating tubular inflammation and injury.^[^^1^^,^^55^^]^ Meanwhile, persistent inflammation and oxidative stress activate the NLRP3 inflammasome, promoting caspase-1-dependent maturation of IL-1β and IL-18 and triggering pyroptosis. These processes establish a vicious cycle in which cardiac dysfunction amplifies renal inflammation, while kidney injury further perpetuates systemic inflammatory responses.^[^^98^^,^^99^^]^ Accordingly, the TLR4/NF-κB pathway and the NLRP3 inflammasome have emerged as promising therapeutic targets within the heart–kidney axis, and pharmacological inhibition of these inflammatory pathways may mitigate sepsis-associated multiple organ injury.

In addition to inflammatory signaling, dysfunction of the heart–kidney axis is also driven by profound hemodynamic disturbances.^[^^100^^]^

Cardiorenal interaction in sepsis (Type 5 cardiorenal syndrome) represents a central and clinically challenging feature of SA-AKI.^[^^101^^]^ Early in sepsis, circulating inflammatory mediators and excessive nitric oxide signaling contribute to sepsis-induced myocardial dysfunction (SIMD), resulting in reduced ejection fraction and diminished forward renal perfusion.^[^^102^^,^^103^^]^

However, growing evidence emphasizes the pathogenic role of venous congestion. The kidney, enclosed within a relatively non-compliant capsule, is highly sensitive to elevations in venous pressure. Right ventricular dysfunction or excessive fluid resuscitation may lead to sustained increases in central venous pressure (CVP), which are transmitted retrogradely to the renal venous circulation.^[^^104^^,^^105^^]^ Effective renal perfusion pressure (ERPP) is determined by the gradient between mean arterial pressure (MAP) and RVP. Even modest increases in RVP can promote interstitial edema, compress tubular structures, and impair microvascular perfusion under capsular constraint.^[^^106^^]^ This hemodynamic paradox – characterized by reduced forward flow and increased backward pressure – precipitates abrupt declines in glomerular filtration rate (GFR) and may perpetuate renal dysfunction even after systemic hemodynamics appear stabilized.^[^^107^^]^

### CKD: the fibrotic transition

Progression from AKI to CKD is driven by persistent TEC injury and sustained inflammatory signaling. Injured TECs release profibrotic mediators, including transforming growth factor-β (TGF-β) and connective tissue growth factor (CTGF), promoting fibroblast activation and differentiation into myofibroblasts, which secrete extracellular matrix components such as type I collagen and fibronectin.^[^^108^^]^

Infiltration of alternatively activated (M2) macrophages further accelerates fibrogenesis via platelet-derived growth factor (PDGF) and insulin-like growth factor-1 (IGF-1) signaling.^[^^109^^]^ Progressive interstitial collagen deposition, capillary rarefaction, and chronic hypoxia exacerbate tubular atrophy and mitochondrial dysfunction. This self-perpetuating cycle of injury, inflammation, and fibrosis may continue during apparent clinical recovery from SA-AKI, ultimately leading to glomerulosclerosis and nephron loss, thereby establishing the pathological basis for post-sepsis CKD.^[^^110^^,^^111^^]^

## Diagnosis and Biomarkers

Given the biological heterogeneity of SA-AKI, different biomarkers reflect distinct pathophysiological processes rather than a single disease mechanism. Table 1 summarizes representative biomarkers according to their biological functions and highlights their potential roles in early diagnosis, risk stratification, and prognostic assessment.

**Table 1:** Biomarkers of SA-AKI.

Biomarker category	Biomarker	Sample source	Known biological function	Potential utility in SA-AKI	References
Tubular injury markers	NGAL	Urine, plasma	Chelates free iron released from injured cells and limits hydroxyl radical formation; induces HO-1; released by activated neutrophils and TECs	Rises within 4–6 h after kidney injury Plasma NGAL predicts renal recovery at hospital discharge in SA-AKI; Plasma levels may increase in systemic infection or inflammation without AKI, limiting sensitivity and specificity for differentiating AKI from CKD	^[^^70^^,^^112^^]^
	KIM-1	Urine	Type I transmembrane glycoprotein acting as a phosphatidylserine receptor; upregulated after tubular injury to facilitate clearance of apoptotic/necrotic cells and epithelial repair	Urinary KIM-1 at 24 h predicts SA-AKI (AUC=0.91) Significantly higher urinary levels at 24–48 h in non-survivors	^[^^113^^]^
	L-FABP	Urine	Cytosolic protein transporting free fatty acids to mitochondria/peroxisomes; limits ROS-mediated injury; upregulated during ischemia–reperfusion	Admission urinary L-FABP levels correlate with mortality in SA-AKI Reflects therapeutic response to PMX-HP	^[^^112^^,^^114^^]^
Cell cycle arrest markers	Urinary [TIMP-2]·[IGFBP7]	Urine	TIMP-2 induces p27 expression and IGFBP7 upregulates p53/p21, jointly inhibiting cyclin-dependent kinase complexes and cell-cycle progression	Predicts SA-AKI with an AUC of ∼0.80 and is minimally affected by non-renal organ failure Approved in multiple regions for AKI risk assessment and regarded as a core marker of cell-cycle arrest	^[^^115^^]^
Endothelial injury markers	sTM	Plasma	Endothelial surface thrombin receptor released upon endothelial activation; regulates coagulation and anti-inflammatory balance	Admission plasma sTM independently predicts SA-AKI (AUC=0.758) Positively associated with disease severity and 28-day mortality	^[^^116^^]^
	Angpt-1	Plasma	Stabilizes endothelial barrier function, limits vascular leakage, and maintains microvascular integrity	Lower plasma levels in SA-AKI than in sepsis without AKI Higher Angpt-1 levels associate with reduced AKI risk	^[^^116^^]^
	Angpt-2	Plasma	Antagonizes Angpt-1 signaling, promotes endothelial activation and vascular permeability, and enhances leukocyte adhesion	Elevated plasma Angpt-2 independently predicts SA-AKI and 28-day mortality A high Angpt-2/Tie-2 ratio identifies high-risk SA-AKI phenotypes (e.g., AKI-SP2)	^[^^116^^]^
	Tie-2	Plasma	Endothelial-specific receptor for Angpt-1/2 regulating vascular stability and renal microcirculatory flow	Reduced Tie-2 levels associate with increased SA-AKI risk, particularly in PERSEVERE-II high- or intermediate-risk groups The Angpt-2/Tie-2 ratio may inform microvascular-stabilizing therapies	^[^^116^^]^
	VE-cadherin	Plasma	Transmembrane adhesion protein forming adherens junctions and maintaining endothelial barrier integrity	Each log-unit increase in plasma VE-cadherin is associated with a 6.44-fold higher risk of RRT in SA-AKI	^[^^116^^]^
	ICAM-1	Plasma	Endothelial adhesion molecule facilitating leukocyte adhesion and transmigration; involved in inflammatory cascades	Adjunct marker of endothelial injury; plasma levels are higher in SA-AKI than in sepsis without AKI and correlate with renal microcirculatory impairment	^[^^116^^]^
	VCAM-1	Plasma	Endothelial adhesion molecule facilitating leukocyte adhesion and transmigration; involved in inflammatory cascades	Elevated plasma VCAM-1 reflects endothelial activation and the extent of renal interstitial inflammation in SA-AKI	^[^^116^^]^
Inflammatory regulatory markers	IL-6	Plasma	Pleiotropic cytokine regulating acute-phase responses and systemic inflammation in sepsis	Admission IL-6 predicts SA-AKI development in severe sepsis Levels correlate with AKI stage and number of organ failures	^[^^117^^]^
	IL-10	Plasma	Anti-inflammatory cytokine inhibiting NF-κB/MAPK signaling and modulating endothelial cell-cycle regulation	Elevated IL-10 independently associates with reduced risk of SA-AKI (OR=0.04, 95%CI=0.01–0.21) Combined with SCUBE2 improves 30-day survival prediction	^[^^118^^]^
	MCP-1	Plasma	Recruits monocytes/macrophages to inflammatory sites and amplifies local inflammation	Higher plasma levels in SA-AKI than in sepsis without AKI Correlates with renal interstitial macrophage infiltration and tubular injury severity	^[^^117^^]^
	sTREM-1	Urine, plasma	Myeloid cell activation receptor selectively involved in infection-related inflammation	Urinary sTREM-1 at ICU admission predicts 48-h SA-AKI (AUC=0.922) Diagnostic AUCs for plasma and urine are 0.794 and 0.707, respectively	^[^^70^^,^^119^^]^
Non-coding RNA markers	homologous to mmu_circ_26986	Plasma, renal tissue	Circular RNA acting as a sponge for miR-29b-1-5p, upregulating PAK7 and inhibiting TEC apoptosis	Differentiates SA-AKI from sepsis without AKI (AUC=0.866) Positively correlates with sCr and is higher in KDIGO stage 2 than stage 1 Potential therapeutic target of the miR-29b-1-5p/PAK7 axis	^[^^120^^]^
Lipid metabolism–related markers	PLTP	Plasma	Regulates lipoprotein metabolism; neutralizes and facilitates clearance of LPS; suppresses inflammatory cascades and preserves endothelial redox balance	Plasma PLTP activity at 24 h predicts SA-AKI and MAKE30 (AUC=0.87); Activity >209.5 pmol/(L·well) associates with reduced MAKE30 incidence; Recombinant human PLTP improves renal function and survival in SA-AKI models	^[^^121^^]^
Endothelial protective markers	SCUBE2	Plasma	Endothelium-derived glycoprotein maintaining vascular barrier integrity; VEGF co-receptor promoting vascular repair	Higher plasma levels in survivors than non-survivors Positively correlates with IL-10 and inversely with APACHE II score, CRP, and MCP-1 Low levels independently associate with increased mortality	^[^^118^^]^
Sepsis risk stratification markers	PERSEVERE-II biomarker panel (IL-8, HSP70, CCL3, CCL4, GZMB, IL-1α, MMP8 plus platelet count)	Plasma	Integrates pathways related to inflammatory activation, stress response, cytotoxicity, and tissue injury	Stratifies SA-AKI into low-, intermediate-, and high-risk groups with differing AKI incidence and RRT requirement Combined with endothelial markers (e.g., Tie-2, Angpt-2) improves 30-day mortality prediction (AUC=0.90)	^[^^116^^,^^122^^,^^123^^]^

AKI, Acute kidney injury; AKI-SP2, Acute kidney injury subphenotype 2; Angpt-1, Aangiopoietin-1; Angpt-2, Angiopoietin-2; APACHE II, Acute physiology and chronic health evaluation II; AUC, Area under the curve; CCL3, C—C motif chemokine ligand 3; CCL4, C—C motif chemokine ligand 4; CI, Confidence interval; CKD, Chronic kidney disease; CRP, C-reactive protein; GZMB, Granzyme B; HO-1, Heme oxygenase-1; HSP70, Heat shock protein 70; ICAM-1, Intercellular adhesion molecule-1; ICU, Intensive care unit; IGFBP7, Insulin-like growth factor-binding protein 7; IL-1α, Interleukin-1 alpha; IL-6, Interleukin-6; IL-8, Interleukin-8; IL-10, Interleukin-10; KDIGO, Kidney disease: improving global outcomes; KIM-1, Kidney injury molecule-1; L-FABP, Liver-type fatty acid-binding protein; MAKE30, Major adverse kidney events at 30 days; MAPK, Mitogen-activated protein kinase; MCP-1, Monocyte chemoattractant protein-1; miR-29b-1–5p, microRNA-29b-1–5p; MMP8, Matrix metalloproteinase-8; mmu_circ_26986, *Mus musculus* circular RNA 26986; NF-κB, Nuclear factor kappa B; NGAL, Neutrophil gelatinase-associated lipocalin; OR, Odds ratio; PAK7, p21-activated kinase 7; PERSEVERE-II, Pediatric sepsis biomarker risk model II; PLTP, Phospholipid transfer protein; PMX-HP, Polymyxin B hemoperfusion; RRT, Renal replacement therapy; SA-AKI, Sepsis-associated acute kidney injury; sCr, Serum creatinine; SCUBE2, Signal peptide-CUB-EGF-like domain-containing protein 2; sTM, Soluble thrombomodulin; sTREM-1, Soluble triggering receptor expressed on myeloid cells-1; TECs, Tubular epithelial cells; Tie-2, Tyrosine kinase with immunoglobulin-like and EGF-like domains 2; TIMP-2, Tissue inhibitor of metalloproteinases-2; VCAM-1, Vascular cell adhesion molecule-1; VE-cadherin, Vascular endothelial cadherin; VEGF, Vascular endothelial growth factor.

## Animal Model of SA-AKI

We systematically summarized the established animal models of SA-AKI with their strengths and limitations in Table 2, and the establishment methods and characteristics in Figure 3.

**Table 2:** Commonly used animal models of SA-AKI: Preparation protocols, strengths and limitations.

Model category	Method	Advantages	Limitations	References	Translational relevance
Single-hit SA-AKI models					
LPS	Intraperitoneal or intravenous injection of purified LPS (e.g., mice 10–15 mg/kg; rats 5–10 mg/kg) to induce endotoxemia	Simple and time-efficient (model established within 24–48 h); high reproducibility; rapid induction of renal inflammation and tubular injury	Models only Gram-negative endotoxemia; cytokine release kinetics differ from human sepsis; requires supraphysiologic LPS doses	^[^^124^^,^^125^^]^	Low
CLP	Laparotomy with ligation of 1/3–1/2 of the distal cecum followed by 1–4 punctures (22–25 G needle)	Considered as the gold standard; mimics polymicrobial intra-abdominal sepsis; reproduces hyperdynamic circulation and multiorgan dysfunction; renal pathology resembles human SA-AKI	Technically demanding; severity varies with ligation length, needle size, sex, and age; limited to intra-abdominal infection	^[^^26^^,^[124], [125], [126], [127], [128]^]^	High
CASP	Placement of a stent in the ascending colon to allow continuous fecal leakage	Sustained pathogen release resembling gut barrier failure; suitable for studying gut–kidney axis	Technically complex; limited standardization; high failure rate due to stent displacement	^[^^129^^]^	Moderate
PCI	Intraperitoneal implantation of fecal or bacterial material	Allows control of contamination severity; models postoperative intra-abdominal infection	Limited use; poor reproducibility; difficulty quantifying pathogen load	^[^^124^^]^	Moderate
Direct bacterial infection	Intravenous or intraperitoneal injection of live bacteria (10^8^–10^10^ CFU)	Enables study of species-specific bacterial effects; controllable inoculum	Represents monomicrobial infection only; high host variability; limited reflection of immune dysregulation	^[^^124^^,^^130^^]^	Low
Two-hit SA-AKI models					
Trauma/hemorrhage + CLP	Initial trauma or hemorrhage followed by CLP after 24–48 h	Mimics “initial insult + infection” scenario; reproduces immunosuppression; renal injury resembles critically ill patients	Complex procedures; very high mortality; poor standardization of timing	^[^[131], [132], [133], [134], [135], [136], [137]^]^	High
I/R + LPS	Bilateral renal artery clamping (30–60 min) followed by LPS injection (5–10 mg/kg)	Models infection superimposed on ischemic kidney injury; emphasizes kidney-specific mechanisms	Requires precise ischemia control; limited representation of multiorgan failure	^[^^138^^]^	Moderate
CKD-based SA-AKI models					
FA-induced CKD + CLP	FA injection followed by CLP after 4–6 weeks	Mimics SA-AKI in CKD with interstitial fibrosis; high SA-AKI incidence	Long induction period; variable fibrosis severity; high post-CLP mortality	^[^^139^^]^	Very high
UNx + adenine diet + CLP	UNx followed by adenine diet and CLP	Controlled CKD progression; good reproducibility; allows study of renal reserve	Weight loss with adenine diet; variability in CKD severity; metabolic CKD only	^[^[140], [141], [142]^]^	Very high
5/6 Nx + CLP	Stepwise resection of renal poles followed by CLP	Stable CKD model with reduced nephron mass; clear SA-AKI pathology	High risk of bleeding and infection; increased postoperative mortality	^[^^143^^,^^144^^]^	Very high

5/6 Nx, 5/6 nephrectomy; CASP, Colon ascendens stent peritonitis; CFU, Colony-forming units; CKD, Chronic kidney disease; CLP, Cecal ligation and puncture; FA, Folic acid; G, Gauge; I/R, Ischemia–reperfusion; LPS, Lipopolysaccharide; PCI, Peritoneal contamination and infection; SA-AKI, Sepsis-associated acute kidney injury; UNx, Unilateral nephrectomy.

**Figure 3:**
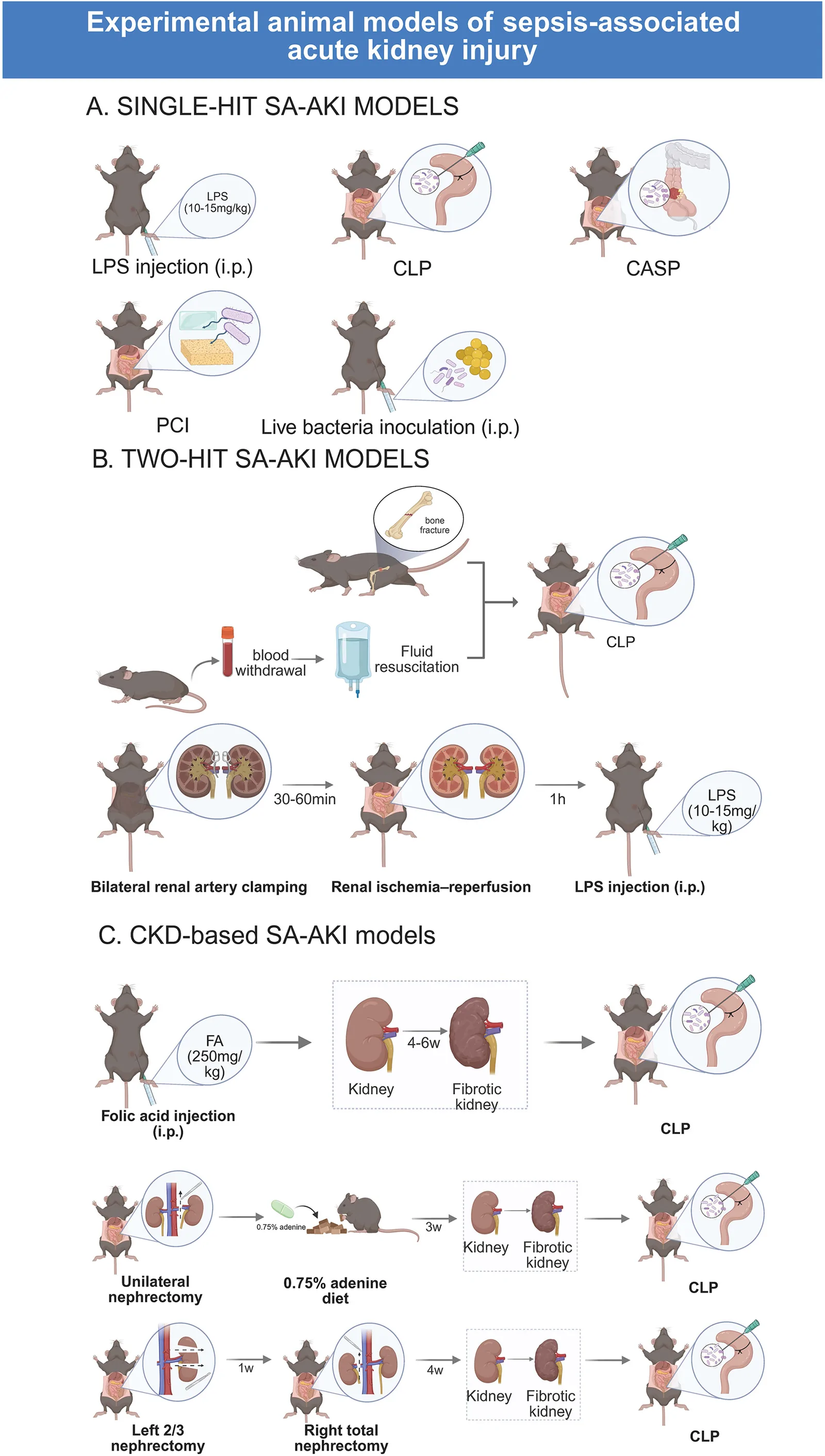
Experimental animal models of SA-AKI. A: Single-hit models: SA-AKI induced by a single insult, including LPS injection, CLP, CASP, PCI, and live bacterial inoculation, mainly used for studying inflammatory mechanisms. B: Two-hit models: Combined insults (e.g., trauma/hemorrhage + CLP or renal I/R + LPS) that mimic clinical complexity and exacerbate renal injury. C: CKD-based models: SA-AKI established on pre-existing kidney injury (e.g., FA, adenine diet, or nephrectomy) to model high-risk conditions. CASP, Colon ascendens stent peritonitis; CKD, Chronic kidney disease; CLP, Cecal ligation and puncture; FA, Folic acid; I/R, Ischemia–reperfusion; LPS, Lipopolysaccharide; PCI, Peritoneal contamination and infection; SA-AKI, Sepsis-associated acute kidney injury.

### Single-factor induction models

The LPS model is widely used because of its technical simplicity and its ability to rapidly induce renal inflammation.^[^^145^^]^ It is particularly suitable for investigating endotoxin-driven inflammatory mechanisms, including activation of the TLR4/NF-κB signaling pathway, as well as for the preliminary screening of anti-inflammatory therapies by evaluating their inhibitory effects on pro-inflammatory cytokines such as TNF-α and IL-6.^[^^146^^,^^147^^]^ However, because it lacks active pathogen replication and fails to reproduce the complex host–pathogen interactions characteristic of clinical sepsis, its translational relevance remains limited.

The cecal ligation and puncture (CLP) model is currently regarded as the gold standard for experimental sepsis because it closely mimics polymicrobial infection and reproduces many pathological features of SA-AKI, including tubular injury, inflammatory infiltration, and multiple organ dysfunction.^[^^126^^,^^148^^]^ Accordingly, it is the most widely used model for investigating the integrated pathophysiology of SA-AKI, including microcirculatory dysfunction, immune dysregulation, and inter-organ crosstalk, as well as for evaluating comprehensive therapeutic interventions such as fluid resuscitation and vasoactive agents.^[^^126^^]^

Colon ascendens stent peritonitis (CASP) and peritoneal contamination and infection (PCI) models generate sustained intra-abdominal infection resulting from disruption of the intestinal barrier and are therefore particularly valuable for studying gut–kidney axis mechanisms, including bacterial translocation and gut microbiota-associated renal injury. However, because of their technical complexity and limited standardization, these models are used relatively infrequently and are generally reserved for studies focusing specifically on abdominal sources of sepsis.

Direct bacterial infection models employ defined bacterial species, such as *Staphylococcus aureus* or *Escherichia coli*, enabling mechanistic investigations of pathogen-specific SA-AKI and evaluation of the combined effects of antimicrobial and renoprotective therapies. Nevertheless, these models are less suitable for reproducing the polymicrobial infections commonly encountered in clinical sepsis.^[^^149^^]^

### Two-hit models

Two-hit models have been developed to better recapitulate the clinical course of critically ill patients by combining an initial insult with subsequent sepsis. The trauma/hemorrhage plus CLP model reproduces the scenario of primary injury followed by secondary infection and successfully mimics the immunosuppressive phase of sepsis.^[^^131^^]^ This model is particularly suitable for investigating immune dysregulation, including macrophage polarization, the protective role of interleukin-10 (IL-10), and the efficacy of immunomodulatory therapies such as granulocyte–macrophage colony-stimulating factor (GM-CSF). Moreover, it provides a valuable platform for exploring risk factors associated with post-traumatic SA-AKI.

The ischemia–reperfusion (I/R) plus LPS model focuses on the synergistic effects of ischemic injury and infection. It is well suited for investigating the interaction between local renal ischemia and systemic inflammation, as well as for evaluating kidney-specific protective interventions, although it does not adequately reproduce the multisystem nature of sepsis.^[^^138^^]^

### SA-AKI models with pre-existing CKD

Experimental models incorporating CKD more closely resemble the clinical population at greatest risk for SA-AKI. The folic acid (FA)-induced CKD plus CLP model establishes renal fibrosis prior to sepsis induction, thereby mimicking SA-AKI superimposed on pre-existing CKD. This model is particularly useful for investigating how reduced renal reserve and chronic fibrosis increase susceptibility to infection-induced kidney injury, as well as for evaluating combined antifibrotic and anti-infective therapeutic strategies.^[^^139^^]^

The unilateral nephrectomy (UNx) combined with an adenine diet and CLP model integrates nephron loss with metabolic injury, enabling investigation of the relationship between diminished renal reserve and susceptibility to SA-AKI, as well as the interaction between metabolic disturbances and sepsis.^[^^140^^,^^141^^]^

The 5/6 nephrectomy (5/6 Nx) plus CLP model produces stable CKD accompanied by clinically relevant complications, including hypertension and proteinuria. It is particularly valuable for investigating how CKD-associated risk factors exacerbate SA-AKI and for evaluating preventive strategies in high-risk populations. Moreover, it represents one of the most widely used models for studying the transition from AKI to CKD.^[^^143^^,^^144^^]^

Despite the availability of numerous experimental models, none fully recapitulates the complexity of human SA-AKI. For example, although the LPS model reliably reproduces sterile endotoxemia and acute inflammatory responses, it fails to capture authentic host–pathogen interactions and the immunological heterogeneity observed in patients, resulting in only low-to-moderate translational relevance. By contrast, the CLP model offers higher translational value owing to its ability to reproduce polymicrobial infection; however, it still does not adequately reflect key clinical features of human sepsis, including antimicrobial treatment, fluid resuscitation, and patient heterogeneity. These limitations may partly explain why many therapeutic strategies that demonstrate efficacy in CLP models ultimately fail in clinical trials. Although the CASP model more faithfully reproduces persistent intra-abdominal infection and continuous bacterial leakage, its technical complexity and limited standardization restrict reproducibility across laboratories. Compared with conventional models, two-hit models and experimental systems incorporating pre-existing CKD more closely reflect the pathophysiological complexity of critically ill patients and therefore possess greater clinical relevance. Nevertheless, successful translation from bench to bedside remains challenging. Beyond the intrinsic limitations of animal models, clinical translation is further hindered by substantial patient heterogeneity, variability in therapeutic timing, and the lack of standardized mechanism-based clinical endpoints. Collectively, these challenges contribute to the persistent gap between promising preclinical discoveries and disappointing outcomes in clinical trials.

Emerging humanized models, including patient-derived kidney organoids, kidney-on-a-chip systems, and multi-organ microphysiological platforms,^[^^150^^]^ provide complementary approaches to conventional animal models. These platforms preserve human-specific genetic and cellular characteristics,^[^^151^^]^ enabling investigation of individualized inflammatory responses, endothelial dysfunction, and drug sensitivity under controlled conditions. However, because they lack mature immune systems and systemic hemodynamic regulation, they cannot yet fully replace *in vivo* models. Instead, they should be regarded as complementary tools for improving the translational relevance of preclinical SA-AKI research.^[^^152^^]^

## Therapeutic Strategies and Drug Development

### Clinical comprehensive interventions: from macroperfusion to microenvironment regulation

Current clinical management of SA-AKI has evolved toward individualized hemodynamic optimization and careful fluid stewardship. Recent randomized clinical trials have challenged the traditional assumption that aggressive fluid resuscitation necessarily improves renal outcomes in patients with sepsis. The CLASSIC trial demonstrated that, following initial resuscitation, a restrictive fluid strategy did not reduce 90-day mortality or confer significant protection against AKI compared with standard care.^[^^153^^]^ Similarly, the CLOVERS trial found no significant difference in 90-day mortality between an early restrictive strategy prioritizing vasopressors and a liberal fluid strategy.^[^^154^^]^ Collectively, these findings suggest that restoration of systemic hemodynamic variables alone is insufficient to reverse SA-AKI, highlighting the pivotal roles of persistent microcirculatory dysfunction, mitochondrial injury, and immune dysregulation in disease progression.^[^^155^^]^ Accordingly, contemporary fluid management has shifted from traditional liberal fluid resuscitation toward a fluid stewardship strategy that emphasizes maintaining fluid balance rather than maximizing fluid administration. Guided by dynamic hemodynamic assessment, balanced crystalloid solutions are preferentially administered in combination with norepinephrine to maintain adequate renal perfusion pressure, while avoiding fluid overload and venous congestion, both of which may further compromise renal function.

Extracorporeal blood purification strategies have also evolved beyond conventional continuous RRT (CRRT). Emerging adjunctive approaches, including cytokine adsorption, high-cutoff membrane hemofiltration, and therapeutic plasma exchange, have been increasingly incorporated with the aim of removing circulating inflammatory mediators and restoring immune homeostasis.^[^^24^^,^^156^^]^ Pharmacological management likewise focuses on minimizing exposure to nephrotoxic agents, optimizing antimicrobial dosing according to renal function and pharmacokinetic principles, and reducing endotoxin burden through adjunctive therapies such as alkaline phosphatase.^[^^157^^]^ In patients with established fluid overload, diuretics may be used to facilitate decongestion when clinically appropriate; however, they should not be considered specific renoprotective therapies for SA-AKI. Evidence supporting vasopressin receptor antagonists in SA-AKI remains insufficient.

### Experimental and translational frontiers: multi-target blockade, metabolic resuscitation, and tissue regeneration

Most mechanism-targeted interventions discussed below remain at the preclinical or early investigational stage. Although several agents have shown renoprotective effects in cellular and animal models of SA-AKI, evidence from dedicated clinical trials remains limited or unavailable.

Given that SA-AKI is a multi-pathway disorder, drug development is increasingly focused on integrated, multi-target strategies aiming to simultaneously block injurious signals and activate endogenous repair programs.

In the realm of anti-inflammatory and immune modulation, therapies targeting key innate immune nodes are gaining attention. TLR4 inhibitors suppress NF-κB activation by interfering with LPS-mediated signaling,^[^^158^^]^ while NLRP3 inflammasome inhibitors block caspase-1–dependent IL-1β/IL-18 release, reducing tubular necroinflammation.^[^^55^^]^ Complement-targeted interventions (e.g., C5a or C5aR antibodies) are also under early clinical investigation for mitigating neutrophil–endothelium–tubule axis-mediated amplification of inflammatory injury.^[^^50^^]^

Mitochondrial protection and metabolic restoration have emerged as major frontiers in SA-AKI research. Given the pivotal role of mitochondrial dysfunction in the pathogenesis of SA-AKI (Section Metabolic reprogramming and mitochondrial dysfunction: “metabolic shutdown” in renal tubular cells), considerable efforts have been devoted to developing mitochondria-targeted therapeutic strategies. Mitochondria-targeted antioxidants, such as mitoquinone (MitoQ) and Szeto-Schiller-31(SS-31), preserve mitochondrial integrity and attenuate oxidative stress, whereas NAD^+^ precursors, including nicotinamide riboside (NR) and nicotinamide mononucleotide (NMN), promote mitochondrial biogenesis through activation of the PGC-1α– Mitochondrial transcription factor A(TFAM) signaling axis, thereby facilitating the restoration of tubular bioenergetics. Although these therapeutic approaches have shown encouraging efficacy in preclinical animal models, their clinical benefits have yet to be convincingly demonstrated in human trials.^[^^159^^,^^160^^]^ Several factors may contribute to this translational gap between experimental studies and clinical application. First, the pharmacokinetic properties of mitochondria-targeted therapeutics remain suboptimal, characterized by limited renal accumulation, inefficient intracellular delivery, and rapid systemic clearance under critical illness.^[^^161^^]^ Second, preclinical studies are generally performed under highly controlled experimental conditions with early therapeutic intervention, whereas patients with sepsis exhibit substantial heterogeneity with respect to the source of infection, immune status, underlying comorbidities, and disease stage.^[^^162^^]^ Third, mitochondrial dysfunction in SA-AKI is highly dynamic and evolves throughout disease progression, making it difficult to define the optimal therapeutic window. Finally, targeting mitochondrial oxidative stress alone is unlikely to be sufficient, as inflammation, endothelial dysfunction, microcirculatory failure, metabolic derangements, and multiple forms of regulated cell death are tightly interconnected and collectively drive disease progression. These limitations suggest that future therapeutic development should emphasize combination treatment strategies, biomarker-guided patient stratification, and optimized drug delivery systems to enhance the translational success of mitochondria-targeted therapies.^[^^161^^]^

Targeting regulated cell death has also emerged as a promising therapeutic strategy for SA-AKI. Increasing evidence indicates that ferroptosis, pyroptosis, and necroptosis are coordinately activated during SA-AKI and collectively contribute to tubular injury. Ferroptosis inhibitors, including Ferrostatin-1 and Liproxstatin-1, protect proximal tubular cells by suppressing lipid peroxidation, whereas necroptosis inhibitors, such as necrosulfonamide, prevent MLKL-mediated plasma membrane disruption and limit the propagation of secondary inflammatory responses, thereby providing novel opportunities for renal protection.^[^^163^^]^

Regenerative medicine and cell-based therapies represent another highly promising therapeutic avenue. Mesenchymal stromal cells (MSCs) and their derived extracellular vesicles (EVs), particularly exosomes, exert potent renoprotective effects through paracrine secretion of reparative mediators, including vascular endothelial growth factor (VEGF), hepatocyte growth factor (HGF), and regulatory microRNAs (miRs). These bioactive factors not only promote tubular regeneration but also modulate macrophage polarization and improve the local immune microenvironment.^[^^164^^]^ Compared with live-cell therapies, exosome-based approaches offer several practical advantages, including lower immunogenicity, greater storage stability, and amenability to bioengineering, making them attractive next-generation therapeutic platforms.^[^^164^^]^

In addition, modulation of autophagy and circadian rhythms has recently attracted increasing attention as an emerging therapeutic strategy for maintaining renal cellular homeostasis. Pharmacological agents that enhance autophagic flux, such as rapamycin and its analogs, facilitate the clearance of damaged mitochondria and aggregated proteins, thereby preserving cellular integrity. Meanwhile, circadian rhythm modulators have been shown to synchronize renal metabolic oscillations and enhance resistance to inflammatory and ischemic insults,^[^^165^^]^ providing a theoretical basis for precision medicine and chronotherapy in SA-AKI.

## Conclusions and Perspectives

Research on SA-AKI has shifted from a traditional “circulatory failure” perspective to a “cellular dynamics and immune dysregulation” framework. This review systematically summarizes the definition, epidemiology, multidimensional pathophysiology, and experimental modeling of SA-AKI, highlighting the central roles of uncontrolled inflammation, microcirculatory dysfunction, metabolic reprogramming, and programmed cell death. The 2023 ADQI consensus provides a standardized framework for clinical subphenotyping and early intervention. Although novel biomarkers, such as [TIMP-2]·insulin-like growth factor-binding protein 7 (IGFBP7) and neutrophil gelatinase-associated lipocalin (NGAL), show promise for early warning, and extracorporeal blood purification demonstrates preliminary efficacy in modulating the cytokine storm, clinical translation remains limited and therapeutic options are still largely single-targeted.

Consistent with the 2023 ADQI consensus, future studies should recognize SA-AKI as a heterogeneous syndrome and prioritize biologically informed subphenotyping and precision-oriented intervention.

## Acknowledgments

Figures in this review were created with BioRender.com.

## Funding

This work was supported by the Non-communicable Chronic Diseases-National Science and Technology Major Project (Nos. 2026ZD0555500 and 2026ZD0555502), Natural Science Foundation of Shandong Province (ZR2023YQ068), and Medical Science and Technology Project of Shandong Province (202619010420).

## Ethics Statement

Not applicable.

## Conflict of Interest

The authors declare that they have no known competing financial interests or personal relationships that could have appeared to influence the work reported in this paper.

## Data Availability

The datasets generated during and/or analyzed during the current study are available from the corresponding author upon reasonable request.

## CRediT authorship contribution statement

**Mengfei Lu:** Writing – original draft, Data curation, Conceptualization. **Qi Zhang:** Writing – review & editing, Validation. **Mingmin Pang:** Writing – review & editing, Validation. **Haoyu Wang:** Writing – review & editing, Conceptualization. **Miaosa Zhang:** Writing – review & editing, Conceptualization. **Yihui Li:** Writing – review & editing, Supervision, Conceptualization. **Hao Wang:** Writing – review & editing, Writing – original draft, Supervision, Conceptualization.
